# Supplementary material for: DMRforPairs: identifying Differentially Methylated Regions between unique samples using array based methylation profiles
Source: BMC Bioinformatics. 2014 May 15;15:141. doi: 10.1186/1471-2105-15-141 (PMC4046028; doi:10.1186/1471-2105-15-141)
Supplement: Additional file 2 — DMRforPairs output for the comparison of A431-MCF7 and NA17018-NA17105. Please start from the HTML files in each folder. Available via the BMC Bioinformatics website. [file 1471-2105-15-141-S2.zip › 1394847754114233_MOESM2_ESM/A431_MCF7/figures/26007.pdf]

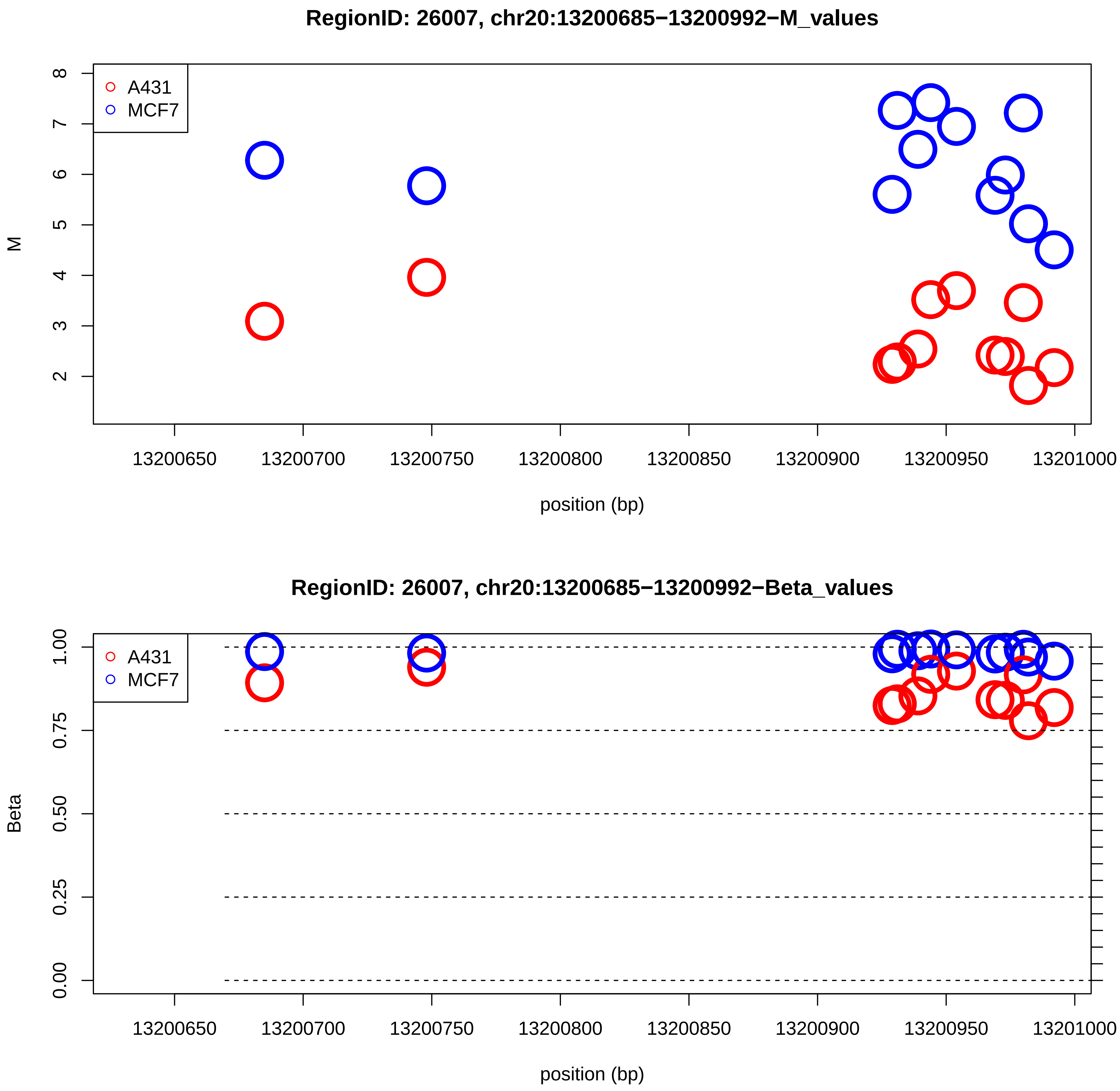

RegionID: 26007, chr20:13200685−13200992−M_values
A431
MCF7
M
8
7
6
5
4
3
2
13200650
13200700
13200750
13200800
13200850
13200900
13200950
13201000
position (bp)
RegionID: 26007, chr20:13200685−13200992−Beta_values
A431
MCF7
Beta
1.00
0.75
0.50
0.25
0.00
13200650
13200700
13200750
13200800
13200850
13200900
13200950
13201000
position (bp)

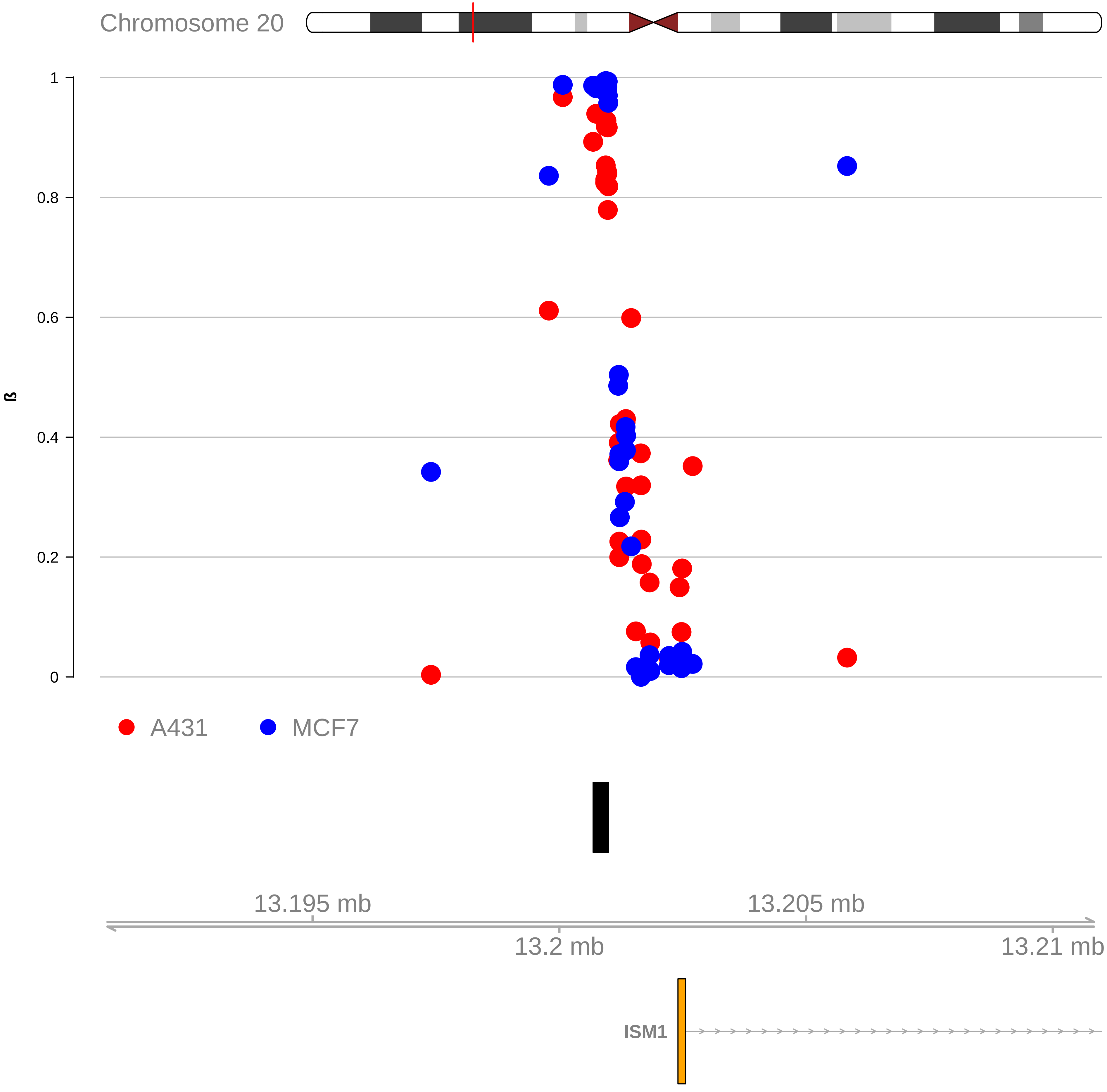
Chromosome 20
1
0.8
0.6
0.4
0.2
0
ß
A431
MCF7
13.195 mb
13.205 mb
13.2 mb
13.21 mb
ISM1
